# Coronary Artery Aneurysm Presenting as Non-ST Elevation Myocardial Infarction

**DOI:** 10.7759/cureus.1436

**Published:** 2017-07-06

**Authors:** Murtaza Sundhu, Mehmet Yildiz, Bilal Saqi, Bilal Alam, Sidra Khalid, Emad Nukta

**Affiliations:** 1 Internal Medicine Residency, Fairview Hospital, Cleveland Clinic, USA; 2 Cardiology, Fairview Hospital, Cleveland Clinic, USA

**Keywords:** coronary artery aneurysm, nstemi, chest pain, myocardial infarction

## Abstract

Coronary artery aneurysms are rare in the general population. There are no randomized control trials to guide the therapy at this moment. We present a case of a 52-year-old male who was recovering from addiction and was sober for past five years. He came to the hospital with typical chest pain. There were ST segment depressions in leads III and AVF. The second troponin was found to be elevated. The impression was non-ST-segment elevation myocardial infarction. He was started on subcutaneous enoxaparin and underwent left heart catheterization which revealed dilated ectatic coronary arteries with aneurysmal dilatation. In addition, there was sluggish blood flow and several blood clots mainly in the left circumflex artery. No intervention was performed and the patient was started on heparin drip which was transitioned to warfarin on discharge. The echocardiogram revealed an ejection fraction of 35% with anterior and inferoseptal wall dyskinesia. Echocardiogram at one-year follow-up showed improved ejection fraction of 50% with similar wall dyskinesia. Coronary artery aneurysms are treated with medical management with or without invasive approach. Invasive management is conducted in people with stenosis and can be achieved by coronary artery bypass graft or covered stents.

## Introduction

Non-ST segment myocardial infarction (NSTEMI) is diagnosed when the patient presents with chest pain and elevated cardiac markers with or without ischemic changes in the electrocardiograph (ST segment depression or T wave inversion). The incidence of NSTEMI has increased when compared to ST-segment elevation myocardial infarction [1]. Coronary artery aneurysm (CAA) is defined as localized dilation (more than 1.5 times) compared to the rest of the coronary arteries [2] and classified based on the shape as saccular or fusiform. The incidence of CAA ranges from 0.3% to 5.3% in the general population with a mean incidence of 1.65% by the pooled analysis [3]. Destruction of the tunica media is the reason for the formation of coronary artery aneurysms [4]. Right coronary artery is the most commonly involved artery followed by left anterior descending or left circumflex artery (different studies have different incidences) [5]. Left main artery or triple vessel involvement is rare [5]. We present a case of triple vessel CAA secondary to atherosclerosis and remote cocaine abuse. Informed consent statement was obtained for this study.

## Case presentation

**Figure 1:**
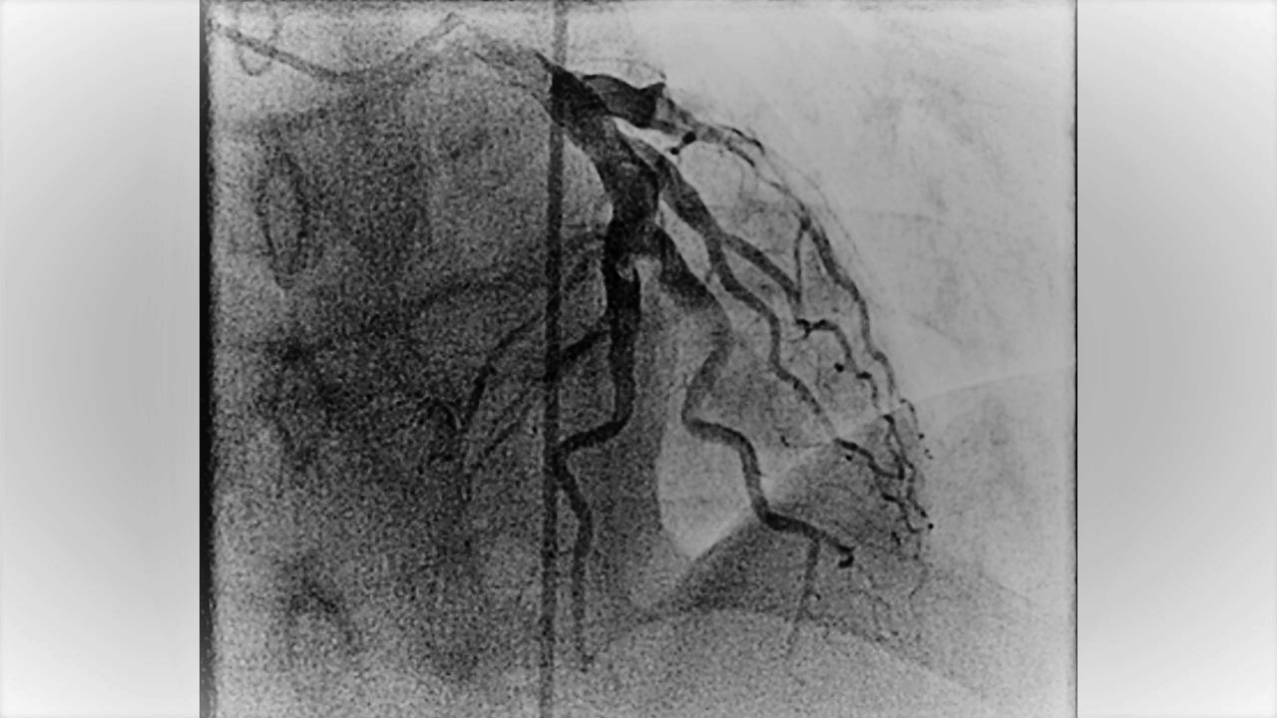
Figure showing the angiography of left anterior descending artery

**Figure 2:**
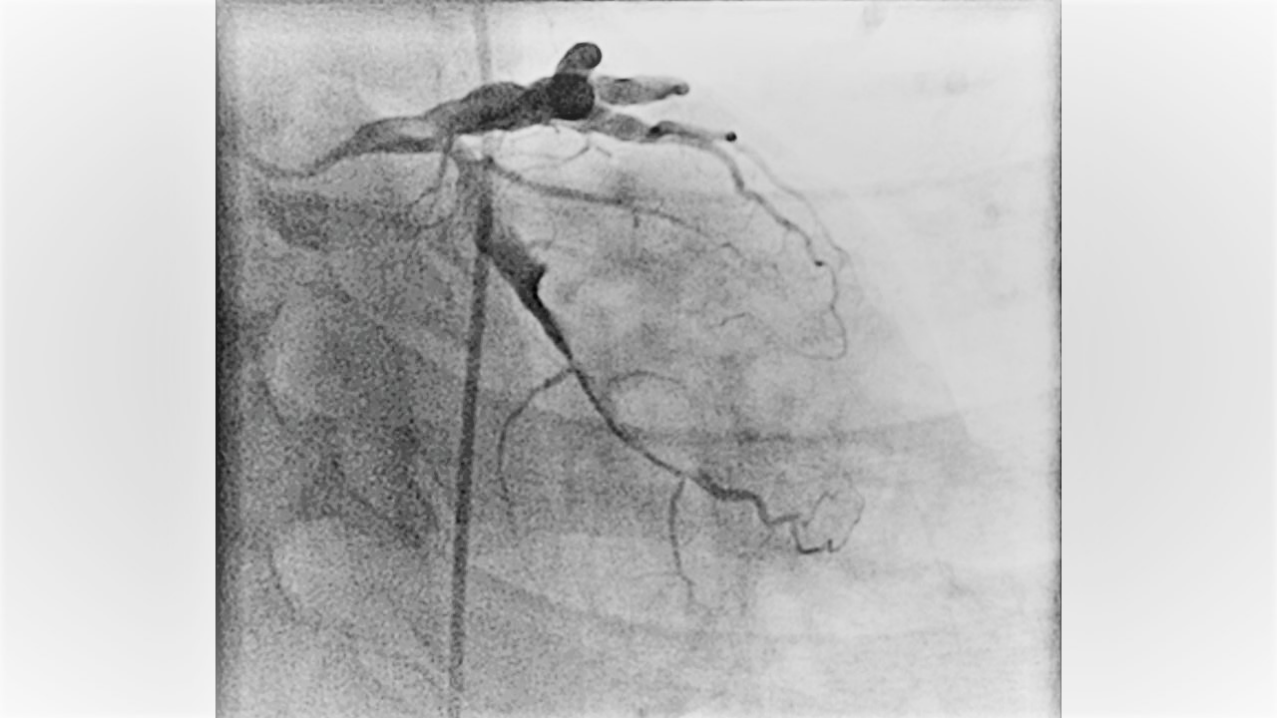
Figure showing the angiography of left circumflex artery

**Figure 3:**
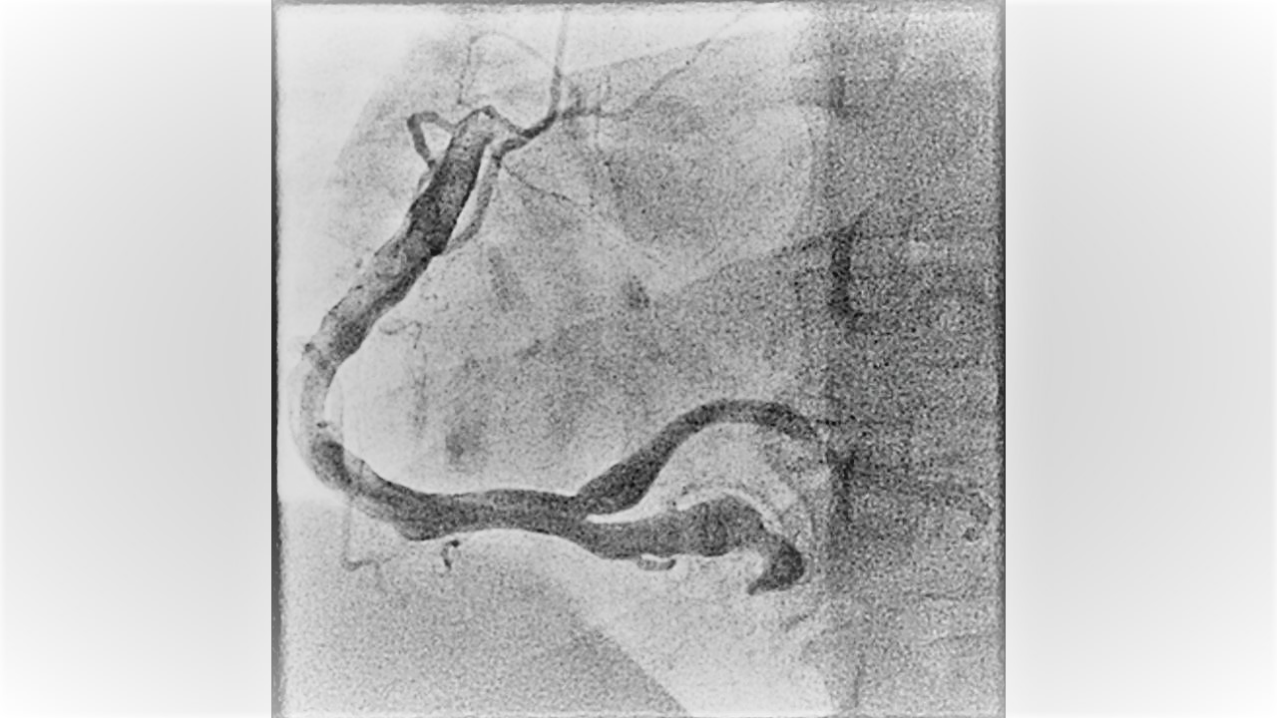
Figure showing the left angiography of right coronary artery

A 52-year-old male with past medical history of migraine, active smoker (with 40 pack year smoking history) and recovering from drug addiction (used cocaine, heroin and prescription opioids) but has been sober for five years now, who presented to the hospital with typical chest pain which was substernal in location with radiation to the jaw and left arm associated with nausea. The chest pain started when he was doing physical exertion. He mostly eats fast food and also has a family history of coronary artery disease at an early age (father had myocardial infarction at age of 40). Complete blood count and comprehensive metabolic profile were within normal limits. The electrocardiogram revealed ST segment depressions in leads III and AVF. Initial troponin was negative. He was admitted to the observation unit to rule out acute coronary syndrome. Subsequent troponins were elevated and he was diagnosed with non-ST elevation myocardial infarction. He was started on subcutaneous enoxaparin in therapeutic dose and transferred to the coronary care unit. He underwent left heart catheterization the next day that revealed dilated ectatic coronary arteries with aneurysmal dilatation, in addition to sluggish flow and several clots mainly in the left circumflex artery (Figure 1-2-3 and Video 1). No intervention was done. He was anticoagulated with intravenous heparin infusion which was transitioned to warfarin. Of note, his C-reactive protein, sedimentation rate, and autoimmune panel were negative. Transthoracic echocardiogram revealed ejection fraction of 35% with anterior and inferoseptal wall dyskinesia. There were no valvular abnormalities. He was discharged on aspirin, beta-blocker, angiotensin converting enzyme inhibitor and a statin for the medical management of myocardial infarction and warfarin was continued for the coronary artery aneurysms with clots. Echocardiogram at one-year follow-up showed the same wall motion abnormalities but the ejection fraction had improved to 50%. He was continued on warfarin.

**Video 1:** Angiography of coronary arteries

## Discussion

Coronary artery aneurysms (CAA) are rare in the general population. The most common cause of CAA in adults about 50% of the cases is atherosclerosis [6]. The other important causes include inflammatory disorders such as Kawasaki disease in children in which the coronary artery aneurysms are pathognomonic. There could be other causes like connective tissue disorders and trauma; which can be blunt, for example, secondary to a drug-eluting stent placement especially with oversized balloons and high inflation pressures. In addition to these causes, cocaine abusers were shown to have increased incidence of CAA the first time in 2005. [7].

Treatment of CAA is not well established because of the rarity of the condition and there are no randomized controlled clinical trials. Medical management is done for all patients, regardless of the invasive management, which includes angiotensin II receptor antagonist which prevents the progression of the CAA although there is no long-term outcome data available. Statins keep atherosclerosis in check. Anticoagulation, especially described with warfarin in literature, is used in patients with larger aneurysm when there is the risk for thrombus formation and embolism [8].

Invasive management was historically done by ligation of an aneurysm and coronary artery bypass graft (CABG). These days covered stent are used in symptomatic patients with associated stenosis. For non-obstructing aneurysms, however, medical management alone is appropriate [8].

Untreated CAA even without stenosis can be complicated by arrhythmias, myocardial infarction or sudden death which is most likely from the thrombus formation and embolization due to sluggish blood flow [9]. Other factors that can contribute to the complications are the extent of atherosclerosis, rupture leading to hemopericardium or tamponade and dissection [6].

Our patient had CAA secondary to a combination of cocaine abuse and smoking history leading to atherosclerosis and presented with acute myocardial infarction to the hospital. He already had clots in the coronary arteries and was at a very high risk for further thrombosis and embolism, hence he was anticoagulated with warfarin. Invasive management was not done because the aneurysms were diffuse and present in all the vessels.

## Conclusions

Coronary artery aneurysm should be kept in mind as a possibility of patients presenting with myocardial infarction, especially the patients with a history of cocaine abuse. Further case series and retrospective studies should be conducted so that we might have a better understanding of the disease pathophysiology and management.
